# Insights into the Classical Genetics of *Clitopilus passeckerianus* – the Pleuromutilin Producing Mushroom

**DOI:** 10.3389/fmicb.2017.01056

**Published:** 2017-06-09

**Authors:** Kate M. J. de Mattos-Shipley, Gary D. Foster, Andy M. Bailey

**Affiliations:** School of Biological Sciences, Life Sciences Building, University of BristolBristol, United Kingdom

**Keywords:** *Clitopilus passeckerianus*, pleuromutilin, antibiotic, basidiomycete, classical genetics

## Abstract

*Clitopilus passeckerianus* is the fungal species responsible for the production of pleuromutilin, a diterpene antibiotic that is gaining in commercial interest. Production of the antibiotic is constrained by the low titers typically obtained from isolates. We therefore set out to investigate the possibility of using classical breeding techniques coupled with genetic manipulation as a means to develop such fungi. We show that the original production strain of *C. passeckerianus* is able to fruit under laboratory conditions, giving viable haploid meiotic basidiospores. The derived progeny displayed the typical physiological and genetic characteristics of a tetrapolar mating system. The monokaryon haploids produced pleuromutilin and haploid lines were amenable to genetic manipulation. Together this shows that the basic requirements for a classical breeding approach are present and the tools required to undertake directed genetic engineering on haploid strains are available, demonstrating that strain improvement may be feasible in this fungus.

## Introduction

*Clitopilus passeckerianus* is the basidiomycete fungus used to commercially produce pleuromutilin, a natural product from which all of the pleuromutilin class of antibiotics are derived. Pleuromutilin was first discovered over 60 years ago during the golden era of antibiotic discovery (Kavanagh et al., 1951), but it was not until the end of the 20th century that derivatives were developed for commercial use. The first two derivatives, tiamulin and valnemulin, have now been used in veterinary medicine for several decades (Poulsen et al., 2001) and in 2007 retapamulin, the first pleuromutilin antibiotic for use in human therapeutics, was approved (Butler, 2008). Most recently, another pleuromutilin derivative called lefamulin has been developed by Nabriva Therapeutics, and is in Phase 3 clinical trials for the treatment of moderate to severe CAPB (community-acquired bacterial pneumonia), and if approved, could become the first pleuromutilin antibiotic for systemic oral administration in humans. Pleuromutilins show no cross-resistance with other antibiotics, such as the macrolides (Waites et al., 2016), and this lack of cross resistance means pleuromutilin derivatives are also the focus of research into novel treatments for multi-drug resistant (MDR-) and extensively drug resistant (XDR-) tuberculosis (Ling et al., 2014; Dong et al., 2015). If pleuromutilin derivatives are to be deployed as affordable mass market antibiotics, there is a need to increase the production capacity of the system, both by optimization of the culture conditions and by genetic strain improvements to the producing fungus. The latter has proven to be immensely valuable for production of other antibiotics, for instance titers of penicillin have been increased by several 1000-fold by such means, making it a cheap commodity chemical (Jami et al., 2010).

The urgent need for new antibiotics (WHO, 2014) means that *C. passeckerianus* could be an increasingly important medicinal species. *C. passeckerianus* is also representative of the underexploited basidiomycetes, a phylum of fungi which are known to display various beneficial medicinal properties, including not only antibacterial, but also anticancer, antiviral, antiparasitic, immunomodulatory, cardioprotective, and hepatoprotective (de Mattos-Shipley et al., 2016). One of the main challenges in exploiting the diverse natural products produced by basidiomycetes, is their complex lifestyles and genetics. Approximately 90% of basidiomycetes are heterothallic, meaning that they must mate with a compatible partner to complete their lifecycles (Raper, 1966). The typical basidiomycete lifecycle involves the germination of basidiospores into monokaryotic mycelia, which after mating with another monokaryon will form stable dikaryotic mycelia capable of fruiting. The genetic basis for compatibility involves pairs of homeodomain transcription factors, and pheromones and receptors. For approximately 60% of heterothallic basidiomycetes these are located at two separate loci; the HD (homeodomain) locus and the PR (pheromone/receptor) locus, in what is known as a tetrapolar system. For bipolar species, either the two loci are linked, or only one locus determines sexual compatibility (Moore et al., 2011; Kües, 2015).

Various strain improvement techniques for the exploitation of natural products are dependent on an understanding of the basic biology, genetics and lifecycle of the producing organism. For example, random mutagenesis, the approach used very successfully for the development of penicillin producing strains (Parekh et al., 2000), requires there to be no genetic redundancy and is best applied to species that produce haploid uninucleate single-celled spores for ease of mutagenesis. The mushroom-forming Agaricomycetes rarely make asexual spores, and with their dominantly dikaryotic lifestyle, mycelial-derived protoplasts are often genetically complex so not an ideal starting material for conventional strain improvement. Breeding programs are occasionally used for strain improvement, and this requires an understanding of the sexual lifecycle of the producing organism and the ability to trigger mating and subsequent fruiting under controlled laboratory settings.

Whilst others have focused on improvements to the media formulations for pleuromutilin production, or using mutagenesis to identify isolates with higher titer (Papa et al., 2006) these have not had the degree of success typically seen with ascomycete fungi, or indeed streptomycete bacteria. The genes responsible for pleuromutilin biosynthesis have recently been isolated and shown to be present together as a gene cluster which is common for secondary metabolite pathways in fungi. Overexpression of the genes in *C. passeckerianus* to increase antibiotic titer had limited success, although refactoring of the regulatory elements and heterologous expression of the genes met with considerable success in *Aspergillus oryzae* (Bailey et al., 2016). In a complementary approach, we sought to determine whether a classical genetics approach might also be feasible for *C. passeckerianus*. Previous work has identified production of pleuromutilin not just from strain DSMZ 1602/ATCC 34646, but also from a range of other isolates within the broader *Clitopilus* genus (Hartley et al., 2009). These isolates typically display a range of growth characteristics and pleuromutilin production capabilities, so have the biodiversity that is usually regarded as a prerequisite for breeding-based improvement programs.

We therefore set out to gain an insight into the biology and classical genetics of *C. passeckerianus*, with particular focus on the production of pleuromutilin.

## Materials and Methods

### Fungal Strain and Growth Conditions

*Clitopilus passeckerianus* strain ATCC 34646 was grown routinely on potato dextrose agar (PDA; Sigma, United Kingdom) at 25°C. MMP (5 g/L mycological peptone, 10 g/L malt extract, 20 g/L agar) was used to generate fruiting bodies. PVS (8 g/L rape seed oil, 35 g/L spray dried corn liquor, 15 g/L glucose, 5 g/L calcium carbonate) was used as the seed medium for HPLC analysis of *C. passeckerianus* due to the homogenous growth obtained. CGC (50 g/L glucose, 5 g/L spray dried corn steep liquor and 2 g/L calcium carbonate, pH 6.5) was used for the production of pleuromutilin in liquid cultures.

### Nucleic Acid Isolation and PCR

DNA was isolated using the rapid mini-preparation of fungal DNA method published by Liu et al. (2000). PCRs were then carried out using GoTaq^®^ DNA Polymerase (Promega) according to the manufacturer’s instructions. The amplified fragments were separated by gel electrophoresis. Details of primers used in this work are shown in **Table 1**.

**Table 1:** Primers used in this work.

Target	Primer	Sequence 5′–3′	Product size
GGS-allele 1	GGS5-F	ATTGAGCTCCACCGTGGTCAA	427 bp
	GGS5-R	TGAAAGCAAGCGACTGCTGA	
GGS-allele 2	GGS34-F	CGTTCGGTCTCTGACATCCAA	427 bp
	GGS34-R	GTCCGTGAATGACAGTAGTTA	
Cyclase-allele 1	Cyclase5-F	ATACATGACCATCGCTCCAAGT	1042 bp
	Cyclase5-R	GTTGGCGTTGCTGATATCTGC	
Cyclase-allele 2	Cyclase34-F	GATGTATCATGGCTGATGATG	979 bp
	Cyclase34-R	GGAGTGCCTTCCAAGCTGCTC	
P450-2-allele 1	P450-2_5F	GCCTTTAAGTTGCTCATTAGC	228 bp
	P450-2_5R	GTGAGGACGCGTTCACCAT	
P450-2-allele 2	P450-2_libF	GCCTTCAAGCTGCTCATAAGT	228 bp
	P450-2_libR	GTGAGGACGCGTTCTCCGT	
FDS-allele 1	FDS2-F	TGAGGCTGAGCGATGAAGTTC	341 bp
	FDS2-R	CCGTTACTTGGGGAGAGGGA	
FDS-allele 2	FDS4-F	GACAGGTGAGTTCCTTCGAGA	289 bp
	FDS4-R	AAATGAGGAAGATCTGTGTTC	
Tubulin-allele 1	Tub-T2F	TGCAGTAGCTGGTTCCACCA	201 bp
	Tub-T2R	AGAGTTTTTACATACCTCGAC	
Tubulin-allele 2	Tub-T12F	ATGCAGTAGCCGGTTCCAGCG	246 bp
	Tub-T12R	GGGCTAAAATAGATCAATTGAGCG	
Actin-allele 1	Actin-A2F	TGTCCGAGCTTGACCCTCTCA	361 bp
	Actin-A2R	ATCGTGGTCAGGGACGATCG	
Actin-allele 2	Actin-A4F	TGTCCGAGCTTAACCCTCTTG	361 bp
	Actin-A4R	ATCGTGGTCAGGGACGAACC	

### PEG-Mediated Transformation of *C. passeckerianus*

Plasmids were transformed into *C. passeckerianus* protoplasts as previously described by Kilaru et al. (2009).

### Bio-Assay for Pleuromutilin Production Detection

To estimate pleuromutilin production in the basidiospore-derived lines, plate-based bioassays against *Bacillus subtilis* ATCC6633 were used as previously described by Hartley et al. (2009).

### HPLC Analysis of Pleuromutilin Production

To quantify pleuromutilin yields, strains were grown in PVS for 5 days to produce a seed media, which was then used to inoculate 100 ml of CGC. Pleuromutilin titers were assessed after 5 days. Three volumes of acetonitrile were added to 1 mL of the fungal culture, which was homogenized using a polytron homogenizer. The sample was centrifuged at 16 000 *g* for 10 min to pellet particulate matter and the supernatant was collected for HPLC analysis. All samples were analyzed using the HP1050 HPLC system. A 260 mm × 4.6 mm C18 column was used with a C18 guard column. The isocratic mobile phase used was 55% acetonitrile with a flow rate of 1 ml/min and a column temperature of 30°C. The absorbance was measured at a wavelength of 205 nm and the retention time for pleuromutilin was approximately 6 min. A pure pleuromutilin standard of 1 mg/ml was used as a control. Titres were quantified against a dilution curve based on a range of pleuromutilin standards.

### Identification of Mating-Type Genes

The genes shown in **Table 2** were used to interrogate *C. passeckerianus* genomic data using the blastn algorithm. The resulting loci were then manually annotated in Artemis (Rutherford et al., 2000), based on gene predictions using the FGENESH program from Softberry (Salamov and Solovyev, 2000) and comparisons to other genes identified from the NCBI database. Sequences were submitted to NCBI and are under accession numbers KY499872-KY499875.

**Table 2:** Genes used to interrogate a *C. passeckerianus* genomic database, for the identification of mating-type loci.

Gene function	Species	Accession number
Homeodomain transcription factors	*Coprinopsis cinerea*	AF126786
	*Schizophyllum commune*	M97181
	*Pleurotus djamor*	AY462112
Pheromone precursors	*Phanerochaete chrysosporium*	BK007881
	*Flammulina velutipes*	HQ630591, HQ630597
	*Coprinopsis cinerea*	AAO17257
Pheromone receptors	*Coprinopsis cinerea*	CAA71962
	*Pholiota nameko*	AB201119
	*Schizophyllum commune*	X77949
MIP	*Pholiota nameko*	AB435542
B-flanking gene	*Coprinellus disseminatus*	AAZ14920
P21 PAK kinase	*Pleurotus djamor*	AY462110

## Results

### The Generation of Monokaryotic Lines

**FIGURE 1:**
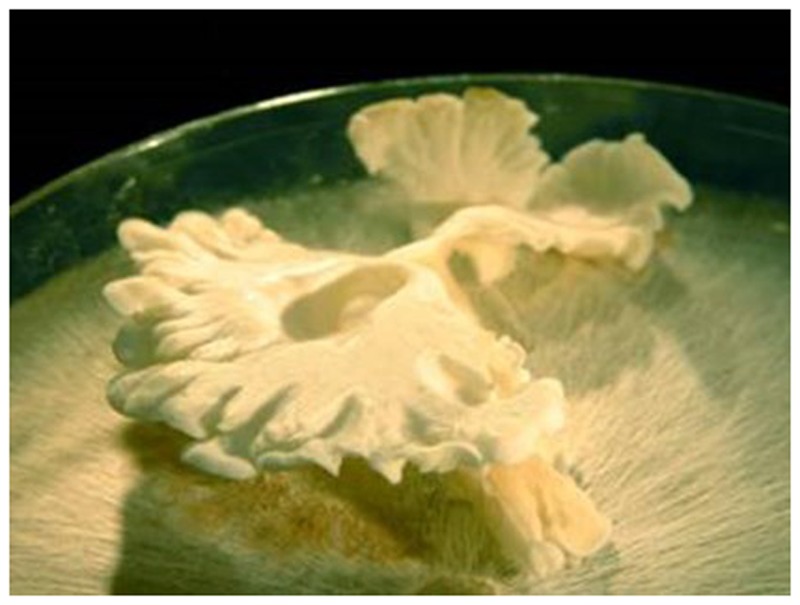
A mature *Clitopilus passeckerianus* fruiting body, 4 weeks after plate inoculation. The brown coloration below the basidiocarp is from the released basidiospores.

Fruiting bodies were generated by growing *Clitopilus passeckerianus* strain ATCC 34646 on MMP plates at 25°C. Approximately 2 weeks post-inoculation, the surface of the agar was entirely covered with hyphal growth and it was possible to observe secondary structures such as hyphal knots or primordia, indicative of fruiting initials. The production of mature, spore producing basidiomes took approximately 4 weeks from inoculation. Microscopy of dissected gill tissues showed the basidia to be tetrasporic, each producing four basidiospores. The basidiospores from mature fruiting bodies (**Figure 1**) were harvested, and when stained with DAPI each spore showed two regions of fluorescence, indicative of a binucleate basidiospore. The spores were diluted and plated onto PDA. Germination occurred immediately and did not require any additional stimulus. The majority of spores were observed to readily germinate suggesting that freshly collected spores have good viability. Hundred such basidiospore-derived colonies (named B1 – B100) were subcultured and maintained on PDA. Initial observations included a variation in growth rate and morphology within the population, but with a readily apparent reduced growth rate when compared to the parental strain, and a noticeable reduction in aerial hyphae, as has been seen for monokaryotic strains of the model basidiomycete *Coprinopsis cinerea* (Kües, 2000).

To provide further supporting evidence that these are indeed haploid lines, allele-specific PCR was performed. Sequence data has been generated previously for a number of genes (Bailey et al., 2016), including actin, β-tubulin, a farnesyl diphosphate synthase (FDS), and three genes from the pleuromutilin gene cluster: the GGS, cyclase and p450-2. The two alleles for each of these genes were aligned and polymorphic regions were identified. Allele specific primers were then designed within these regions, with at least two SNPs located at the 3′ end of the primer, to allow allele specific amplification.

**FIGURE 2:**
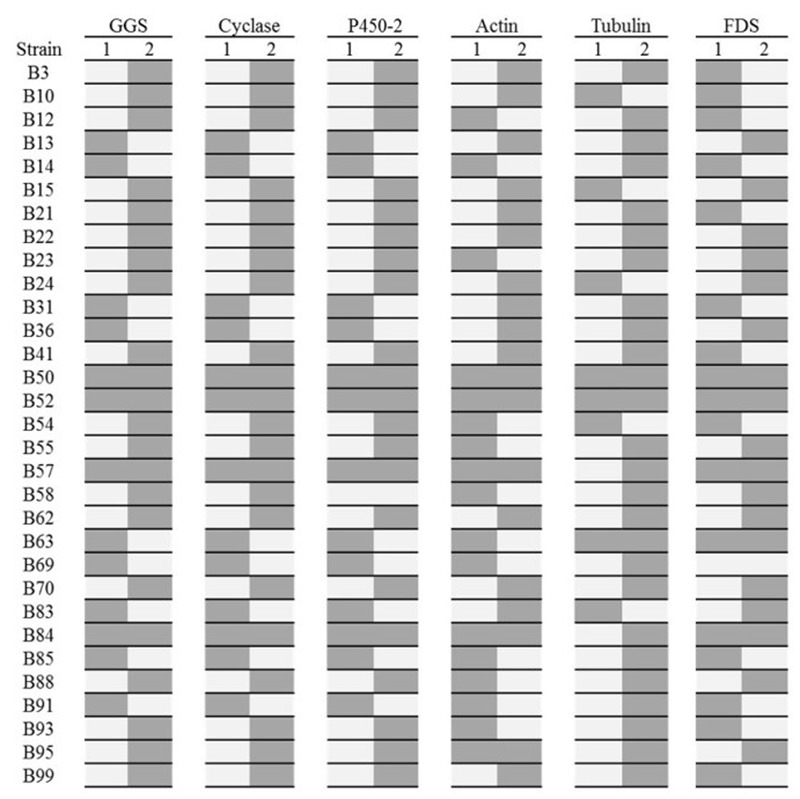
Allele specific PCRs for 31 basidiospore-derived lines. Dark gray denotes the presence of an allele. The six genes analyzed were a GGS, Cyclase and P450-2 from the pleuromutilin gene cluster and actin, tubulin and FDS. The presence of only one allele for all six genes (four loci) for many of the lines suggests they are monokaryotic. Lines B50, B52, B57, B63, and B84 for both alleles at various loci suggesting they are dikaryotic. The identical patterns for the *GGS, cyclase* and *P450-2* demonstrate that no recombination has occurred within this region of the pleuromutilin gene cluster.

For the majority of the lines screened, only one allele could be amplified for each gene (**Figure 2** and Supplementary Figures 1, 2), strongly suggesting that these strains contain only one haploid nucleus, as would be expected if originating from basidiospores. Two strains, B50 and B52, contained both alleles for all genes screened, implying that these strains are dikaryotic and likely to be genetically identical to the parental strain. B50 in particular was noted as having identical morphology to the parental strain. Two further strains, B57 and B84 were positive for both alleles for all of the genes except tubulin, suggesting that these strains may be the product of mating and are homozygous at the tubulin loci. Strain B95 gave amplification products when using both sets of allele-specific primers for actin. However, the primers designed to amplify actin-allele2 were capable of amplifying a product from a plasmid containing actin-allele 1, demonstrating non-specific binding does occurs for this primer/allele combination when the template concentration is high.

Rather unexpectedly, non-random reassortment appears to have occurred for tubulin locus, with 87% of lines containing the tubulin-2 allele. This may be due to the presence of a detrimental mutation in a gene showing genetic linkage to the tubulin-1 allele.

An identical allele-specific pattern for the three pleuromutilin genes in the presumed haploid lines demonstrates that no recombination has occurred within the 10,356 bp region of the cluster spanning the *P450-2* to the *cyclase*. However, different allelic patterns for the different loci (the pleuromutilin gene cluster, actin, tubulin and FDS), shows that karyogamy and reassortment has occurred between unlinked loci to produce genetically distinct progeny.

A morphological characteristic of basidiomycetes often used to distinguish monokaryotic and dikaryotic tissues is the presence of clamp cells, which are involved in maintaining two nuclei per cell in the dikaryotic tissues of many basidiomycetes. Unfortunately, careful microscopic examination of mycelia grown on a range of media, with varying pH, gave no evidence of clamp connection formation in the parental strain. This suggests that *C. passeckerianus* may not produce clamp connections, or not reliably under laboratory conditions, as is known to be the case for some basidiomycetes (Webster and Weber, 2007).

### Pleuromutilin Production in Monokaryotic Lines

**FIGURE 3:**
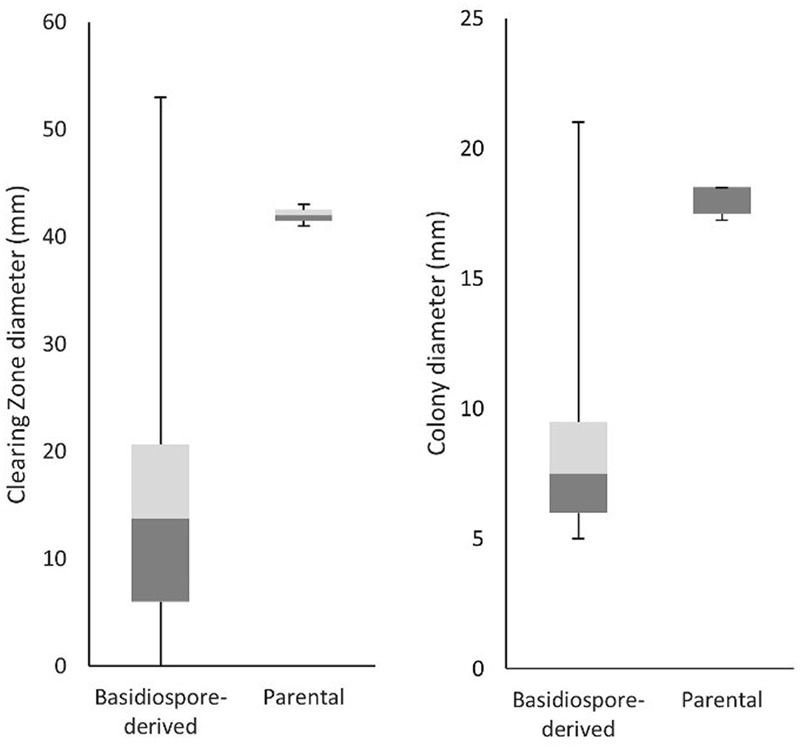
Clearing zone diameters (indicative of pleuromutilin production) and growth rate data for the 100 lines generated from basidiospores. The average clearing zone diameters was much lower for the basidiospore derived lines, although this can be explained by the lower growth rate. A large amount of variation was seen within the population of basidiospore-derived lines.

To determine whether *C. passeckerianus* produces pleuromutilin during the monokaryotic stage of its lifecycle, bioassays were conducted for all 100 basidiospore-derived lines. Two bioassay plates were produced for each strain and the diameters of the inhibition zones and fungal colonies were recorded (Supplementary Figure 3). Inhibition of *B. subtilis* growth was observed for 81 of the 100 strains. The majority produced smaller clearing zones than the wild-type parental strain, with the average clearing zone diameter being 15.7 mm compared to 42 mm. However, most lines also had a far slower growth rate, with the average colony diameter being 8.47 mm compared to 17.5 mm for the parental strain, which may account for the observed reduction in bacterial inhibition. A high level of variation, both in terms of growth rate and bacterial inhibition, was observed within the population of basidiospore lines (**Figure 3**).

**FIGURE 4:**
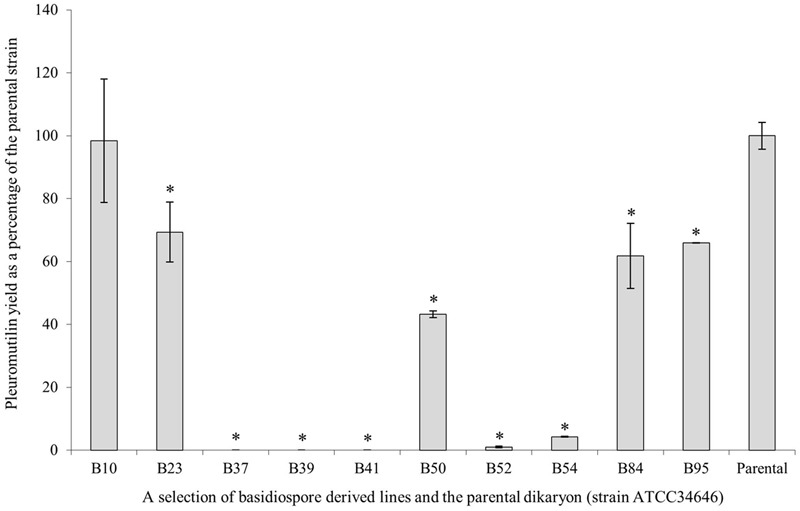
Pleuromutilin yields in liquid cultures for a selection of basidiospore-derived cultures, shown as a percentage of the parental strain, *C. passeckerianus* ATCC 34646. Data presented are averages of two independent biological replicates ±SE. Asterisks indicate those significantly different from the parental strain (*p* < 0.05).

To obtain a quantified measure of pleuromutilin yield in fermentation cultures, which is more relevant to the commercial production of pleuromutilin, 10 basidiospore-derived lines, including three strains thought to be dikaryotic on the basis of morphology and allelic PCR, were grown in production media (CGC). Crude extracts were analyzed by HPLC (**Figure 4**). B37, B39, and B41 did not produce detectable levels of pleuromutilin in liquid culture and a further two (B52 and B54) produced trace amounts. B23, B50, B84, and B95 produced between 42 and 70% of the wild-type strain, and B10 alone had a comparable, if not slightly higher yield, when biomass was taken into account.

### Mating Experiments

In order to gain an estimation of mating frequency between sibling strains, 10 monokaryons, all of which have been screened using allele specific PCR (B10, B12, B13, B15, B21, B22, B63, B70, B88, and B93) were paired in all combinations. For each pairing, the two strains were inoculated to one side of a petri dish (containing 25 ml of PDA) approximately 30 mm apart. These were incubated at 25°C until mycelia contact was established. At this point any changes in morphology or growth rate were noted, and where a clear change had occurred, a section was subcultured until consistent morphology was achieved. As controls, two *Coprinopsis cinerea* mating plates were set up; one with incompatible strains (LN118 and PG78) and one with compatible strains (LN118 and AT8) (Supplementary Figure 4). For the compatible strains, a clear increase in growth rate and aerial hyphae could be observed, starting at the point where the two strains met. A similar phenomenon was observed for a number of the *C. passeckerianus* crosses (Supplementary Figure 4). Of the 45 combinations tested, 13 demonstrated a clear change in morphology and were chosen for further analysis: B10–B15, B12–B88, B12–B93, B13–B88, B13–B93, B15–B21, B15–B22, B15–B88, B15–B93, B21–B93, B22–B63, B22–B88, and B88–B93.

**FIGURE 5:**
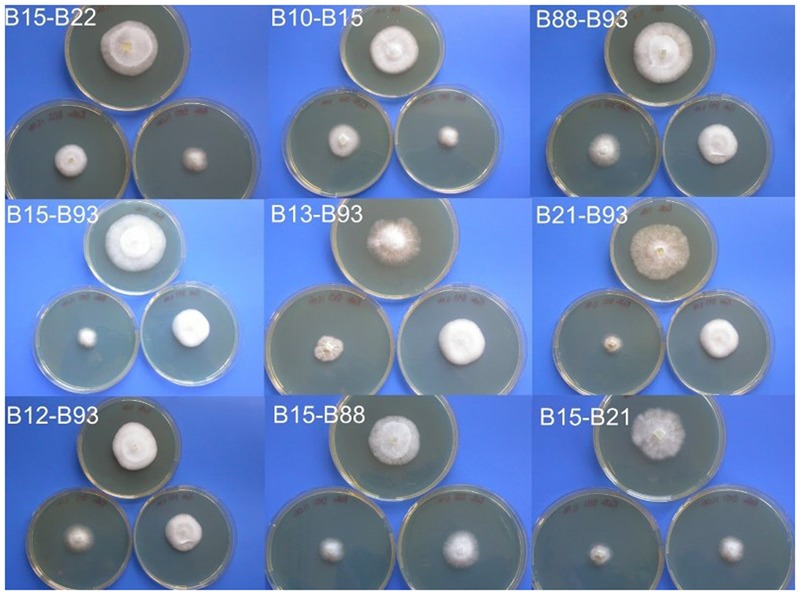
Colonies resulting from crossing basidiospore-derived *C. passeckerianus* strains. Each photograph shows the two mated monokaryotic lines below, with the resulting dikaryon above, demonstrating the faster growth rate and increased aerial hyphae in mated strains. Photographs were taken 6 days after inoculation on PDA.

During the process of subculturing the potential crosses, four pairings (B12–B88, B12–B93, B13–B88, and B22–B63) appeared to segregate back into two colonies with identical morphology to the two originally paired strains, suggesting that these crosses did not produce stable dikaryotic mycelia, possibly due to semi-compatible matings. However, the remaining nine pairings produced stable colonies each with a clearly increased growth rate (**Figure 5**). The frequency of successful matings from these crosses was 20%, which supports a tetrapolar mating system being employed by *C. passeckerianus*.

We were not able to generate mature fruiting bodies from these successful pairings, but this is not unusual amongst sibling-matings in basidiomycetes.

### Identification of Putative Mating Type Loci

Although the observations from sibling crosses support a tetrapolar mating system, this was not fully proven due to the absence of clamp connections and difficulty in triggering fruiting in the resulting crosses. We obtained genome sequence data for *C. passeckerianus* ATCC34646 (unpublished) and this was searched for homologs to the well-defined homeodomain (HD) and pheromone/receptor (PR) mating type loci.

**FIGURE 6:**
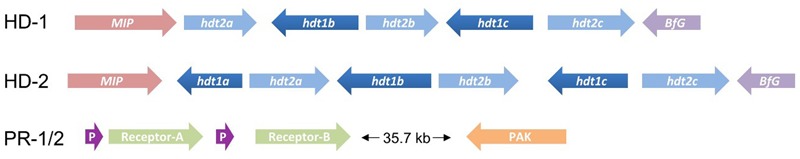
Mating type loci identified for *C. passeckerianus* ATCC34646. The two HD locus alleles differed significantly, with HD-1 missing one of the type 1 homeodomain genes. The two alleles of the pheromone/receptor (PR) locus had identical gene organization.

The database was interrogated using known mating type genes from a range of basidiomycete species, including homeodomain transcription factors, receptors and pheromones, and also genes frequently found flanking, or located near to, mating type loci (see **Table 2** for details). This resulted in the identification of homologs to both known mating type loci previously identified in tetrapolar basidiomycetes, each represented by two idiomorphic alleles on separate contigs. Both alleles of the HD locus are flanked by genes encoding a MIP and BfG, as commonly seen in other basidiomycetes, and outside this region there is very high homology between the two allelic loci. In contrast, within the mating-type locus itself, there are clear differences in gene organization. Homeodomain allele 1 (HD-1) contains two complete pairs of type 1 (*hdt1*) and type 2 (*hdt2*) homeodomain transcription factors, with an additional *hdt2* adjacent to the MIP (**Figure 6**). Homeodomain allele 2 contains three complete pairs of homeodomain transcription factors, although one of the *hdt2* genes (*hdt2b*) appears to have a stop codon within the conserved homeodomain motif, suggesting that this gene is not functional. It is worth noting that that fungal homeodomain genes are highly divergent, with the predicted proteins typically sharing only 5–15% sequence homology at the protein level (Supplementary Table 1), those identified in this analysis are typical of this, with little overall sequence identity but they all contain the homeodomain domain motifs recognized by the conserved domain database of NCBI.

The two alleles of the putative pheromone/receptor (PR) locus have identical genetic organization. Two receptors could easily be identified, approximately 36 kb from a p21-activated kinase (PAK), a gene commonly associated with this locus. Adjacent to each receptor, a small open reading frame could be identified which encodes a predicted protein including the C-terminal CAAX motif (where C is cysteine, A is aliphatic, and X is any residue) typical of pheromone precursors. This motif is known to be required for the C-terminal cleavage and farnesylation to produce the mature pheromone (Raudaskoski and Kothe, 2010). Such pheromone precursors are notoriously difficult to identify, due to their small size and overall lack of conserved sequence, so RNAseq data would be needed to confirm the identity of these genes. Far less sequence divergence is seen between the alleles of the PR locus, with both receptors having over 94% sequence identity with their homolog from the other allele. Also worth noting was the presence of multiple prenyltransferase genes located between the PAK and the receptors, which may be responsible for the farnesylation of the immature pheromone precursors.

### Transformation of a Monokaryon

**FIGURE 7:**
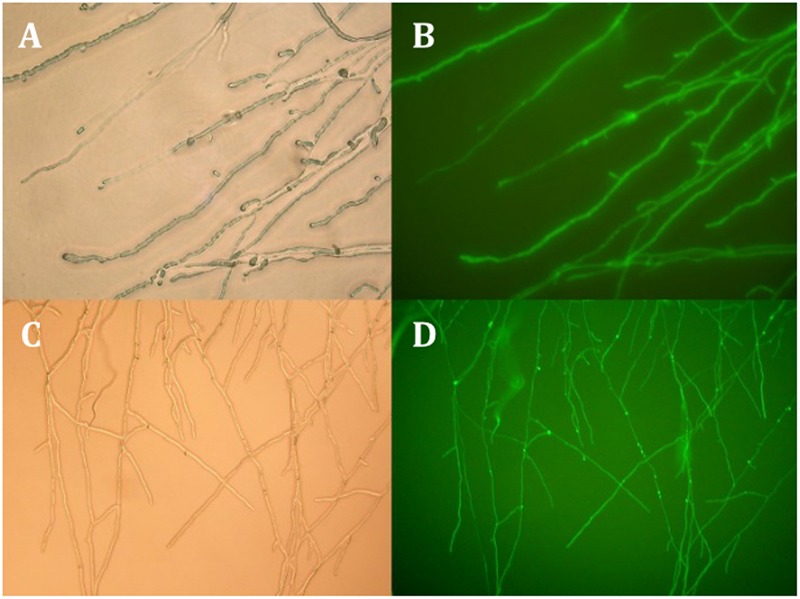
White light and fluorescent micrographs of two B36 transformants exhibiting GFP fluorescence. The top two panels **(A,B)** show a strain that resulted from a co-transformation using pMhphi004, which confers hygromycin resistance and pGFPi004, which contains a GFP expression cassette. The lower panels **(C,D)** show a transformant containing pYES-hph-004iGFP; a plasmid that has both the hygromycin resistance and GFP expression cassettes.

Techniques such as mutagenesis and gene disruption are often problematic with dikaryotic basidiomycetes because of genetic redundancy due to two nuclei per cell. They could, however, be employed when working with a monokaryotic line. To investigate the potential of such techniques we tested whether the transformation protocol previously developed for *C. passeckerianus* (Kilaru et al., 2009) was applicable to a monokaryotic strain. Strain B36 was chosen as this strain has been shown to be monokaryotic by PCR, reliably produces pleuromutilin and has a reasonable growth rate. Two transformations were performed using the reporter gene *GFP*: one as a co-transformation using pGFPi004 and the hygromycin resistance plasmid pMhphi004 (Kilaru et al., 2009), and one transformation with pYES2-hph-004iGFP (derived from pGFPi004), which contains both the GFP and hygromycin expression cassettes. Twenty-four hygromycin resistant transformants were obtained using the co-transformation technique, a third of which demonstrated GFP expression, showing that the integration of multiple plasmids by co-transformation is a fairly frequent occurrence in strain B36. That said, a more efficient way of achieving GFP expression was by using the single plasmid pYES2-hph-004iGFP containing both cassettes, which resulted in over 70% of transformants (26 of 37) expressing GFP (**Figure 7**).

To investigate the potential of using gene silencing to study gene function in monokaryotic lines, a construct that has previously been used to silence the pleuromutilin *GGS* (pYES2-hph-GGSantigene) (Bailey et al., 2016) was transformed into strain B36. Fifty hygromycin resistant transformants were obtained and inhibition of *B. subtilis* was measured on bioassay plates to give an indication of pleuromutilin production (Supplementary Figure 5). The average clearing zone diameter was 84.8% of wild-type. Eight transformants demonstrated a reduction of over 35% and for transformant B36-*GGS*-AS-25, no *B. subtilis* inhibition was observed, indicating no detectable antibiotic production. This data suggests that gene silencing could bea viable technique for investigating gene function in a monokaryotic *C. passeckerianus* strain. When compared to the set of 51 dikaryotic transformants generated previously (Bailey et al., 2016), the average clearing zone diameter is similar, being 84.8 and 86.7% of wild-type, respectively. However, only one of the dikaryotic transformants demonstrated a reduction greater than 35%, suggesting that silencing in a monokaryon results in a strong observable phenotype more frequently than in a dikaryon.

## Discussion

The pleuromutilin producing strain of *C. passeckerianus* originally reported by Kavanagh et al. (1951), and which is used in the production of pleuromutilin, has previously been shown to be dikaryotic (Hartley et al., 2009; Bailey et al., 2016). In this work we have also shown that this strain is fertile; fruiting under laboratory conditions and producing binucleate basidiospores that germinate to produce viable progeny. Multinucleate basidiospores are known to be common for basidiomycete species. In some cases, such as the pseudohomothallic *Agaricus bisporus* var. *bisporus*, each spore contains two types of nuclei that are genetically distinct, and the spores will germinate to produce fertile secondary mycelia capable of fruiting (Kerrigan et al., 1993). Far more common for basidiomycete fungi, however, is the heterothallic mating system (Raper, 1966) where multiple nuclei result from the replication of a single haploid nucleus during spore maturation, as is the case for *Agaricus bisporus* var. *burnettii* (Kerrigan et al., 1994). The germination of such spores results in primary monokaryotic mycelium that then requires mating to complete the lifecycle. Monokaryotic mycelia, which has been intensively studied in model species such as *Coprinopsis cinerea*, is characterized by a slower growth rate and relative lack of aerial mycelia compared to the parental strain (Kües, 2000). Initial observations for the basidiopore-derived *C. passeckerianus* lines were indeed consistent with the morphology of genetically distinct monokaryotic lines, where variation has been introduced through karyogamy and reassortment. This was confirmed by an analysis of the allele distribution in these lines, where the vast majority of lines screened had only one allele for each locus tested, and the differing distribution patterns of the genes on each strain demonstrated that reassortment had indeed occurred.

A number of sibling progeny were screened for pleuromutilin production, which was observed both on solid media and in fermentation. Varied levels of pleuromutilin production were recorded, despite no recombination of alleles within the 10,356 bp region of the pleuromutilin gene cluster screened, suggesting that multiple factors beyond the gene cluster itself impact pleuromutilin biosynthesis. The variation observed, both in terms of morphology and pleuromutilin production, means it may be possible to select lines which have better titers and are better suited to growth in fermenters. The production of pleuromutilin by haploid lines also means that it may be feasible to use conventional mutagenesis directly on these strains for strain improvement, approaches that are not generally feasible in dikaryotic strains due to their genetic redundancy. To investigate whether rational strain development via genetic engineering would also be feasible, a monokaryotic strain was transformed using various plasmids. eGFP expression was achieved via co-transformations, where the eGFP and selection marker cassettes were on separate plasmids, showing that it is possible to have integration of multiple plasmids in one transformation step, even when selecting for only one of the plasmids. Higher frequencies of eGFP expression were achieved, however, by using a single plasmid containing both the selection and reporter gene cassettes. Phenotypes consistent with gene silencing were obtained in transformants generated using a plasmid containing an antisense cassette, demonstrating that the comprehensive suite of molecular techniques developed for the parental strain (Kilaru et al., 2009) are applicable for monokaryotic strains.

The reduced growth rate and lower absolute pleuromutilin yields generally seen in haploid strains may limit the development of such strains for commercial pleuromutilin production, but it may be possible to circumvent this by also employing a breeding program to generate improved dikaryotic strains. Thus, the mating system of *C. passeckerianus* was investigated. The monokaryotic strains demonstrated mating frequencies typical of a tetrapolar genetic system, and this was backed up by detailed analysis of the mating loci in this isolate. Mating-type loci homologous to the well-defined homeodomain (HD) and pheromone/receptor (P/R) loci of tetrapolar basidiomycetes were identified and annotated. One of the HD loci identified contained three pairs of types 1 and 2 homeodomain transcription factors, while the second locus was found to be lacking one of the type 1 genes. This is comparable to the archetype *C. cinerea* HD mating type loci, which typically contain three pairs of HD genes. Both P/R loci identified contained two putative pheromone/receptor pairs, which is the case for the well-known tetrapolar species *Ustilago maydis*, although significant variation in the structure and content of this loci is seen in different species. In addition to the mating type genes being homologous to those of model basidiomycete species, their genomic context appears to be conserved, with the homeodomain mating-type genes being typically flanked by genes encoding a MIP and BfG, and the pheromone/receptor genes being located near to a p21-activated kinase. Additionally, the location of the two *C. passeckerianus* mating-type loci on separate and large genomic scaffolds supports the tetrapolar nature of *C. passeckerianus*, with the genomic loci appearing unlinked. In some well-studied bipolar species, such as *Ustilago hordei* and *Microbotryum violaceum*, the two loci are still present but have become tightly genetically linked, effectively acting as a single mating-type locus. Given the previous levels of variation reported in pleuromutilin producing wild-type *C. passeckerianus* strains (Hartley et al., 2009), there may well be enough diversity in wild populations to use as the basis of a breeding program – particularly if out-crossing maintains fertility.

The above findings all suggest that strain improvement for *C. passeckerianus* would be possible by traditional means, but it must also be considered that this might be a slow and random process, and a synthetic-biology approach may deliver improvements more rapidly, as recently shown by Bailey et al. (2016) who expressed the entire pleuromutilin pathway in the heterologous host *A. oryzae*.

## Author Contributions

The initial project was devised by GF and AB. KdM-S designed and carried out the experiments and subsequent analysis, with supervisory support from AB and GF. KdM-S compiled the manuscript with contributions and editing roles supplied by AB and GF.

## Conflict of Interest Statement

The authors declare that the research was conducted in the absence of any commercial or financial relationships that could be construed as a potential conflict of interest.
