# Supplementary material for: Insights into the Classical Genetics of Clitopilus passeckerianus – the Pleuromutilin Producing Mushroom
Source: Front Microbiol. 2017 Jun 9;8:1056. doi: 10.3389/fmicb.2017.01056 (PMC5465285; doi:10.3389/fmicb.2017.01056)
Supplement: Supplementary file 1 [file Data_Sheet_1.DOCX]

Supplementary Material

Insights into the classical genetics of *Clitopilus passeckerianus* – the pleuromutilin producing mushroom

**Kate M. J. de Mattos-Shipley^1^, Gary D. Foster^1^ and Andy M. Bailey^1*^**

*** Correspondence:** Corresponding Author [Andy.Bailey@bristol.ac.uk](mailto:Andy.Bailey@bristol.ac.uk)

# Supplementary Figures and Tables

## Supplementary Figures

**
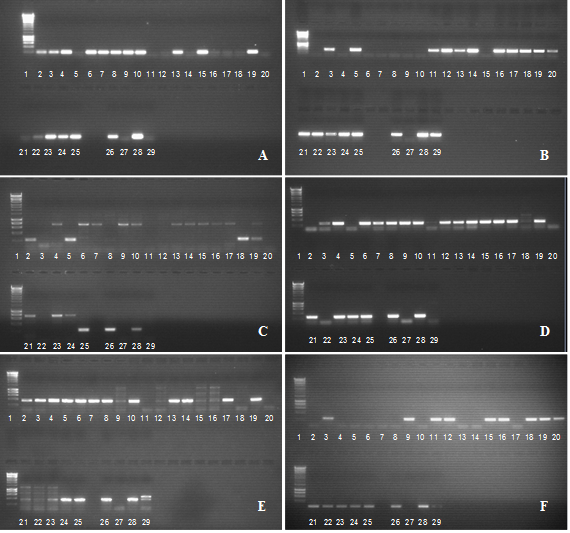
**

**Supplementary Figure 1**: Allele specific PCRs, designed to identify which allele is present in a number of basidiospore derived lines at three separate loci (actin, tubulin and FDS). Lane 1 of all gels contains 5µl of Hyperladder I from Bioline. Lanes 2 – 25 are various basidiospore-derived lines. Lanes 26 - 29 are controls (see below).

A) Actin allele 1. Product size: 361 bp. Controls: WT, H_2_0, Act1 clone, Act2 clone.

B) Actin allele 2. Product size: 361 bp. Controls: WT, H_2_0, Act2 clone, Act1 clone.

C) Tubulin allele 1. Product size: 201 bp. Controls: WT, H_2_0, Tub1 clone, Tub2 clone.

D) Tubulin allele 2. Product size: 246 bp. Controls: WT, H_2_0, Tub2 clone, Tub1 clone.

E) FDS allele 1. Product size: 341 bp. Controls: WT, H_2_0, FDS1 clone, FDS2 clone.

F) FDS allele 2. Product size: 289 bp. Controls: WT, H_2_0, FDS2 clone, FDS1 clone.

**Supplementary Figure 2**: Allele specific PCRs, designed to identify which allele is present in a number of basidiospore derived lines for three of the genes involved in pleuromutilin biosynthesis (the GGS, cyclase and P450-2). Lane 1 of all gels contain 5µl of Hyperladder I from Bioline. Lanes 2-25: PCR reactions for 24 basidiospore-derived lines. Lanes 26-29 are controls (see below). The identical patterns seen in panels A, C, E and B, D, F demonstrate that no recombination has occurred in this region of the pleuromutilin gene cluster.

A) Cyclase-1 allele. Product size: 1042 bp. Controls: WT, H_2_0, Cyc1 clone, Cyc2 clone.

B) Cyclase-2 allele. Product size: 979 bp. Controls: WT, H_2_0, Cyc2 clone, Cyc1 clone.

C) P450-2-1 allele. Product size: 228 bp. Controls: WT, H_2_0, P450-2-1 clone, P450-2-2 clone.

D) P450-2-2 allele. Product size: 228 bp. Controls: WT, H_2_0, P450-2-2 clone, P450-2-1 clone.

E) GGS-1 allele. Product size: 427 bp. Controls: WT, H_2_0, GGS1 clone, GGS2 clone.

F) GGS-2 allele. Product size: 427 bp. Controls: WT, H_2_0, GGS2 clone, GGS1 clone.


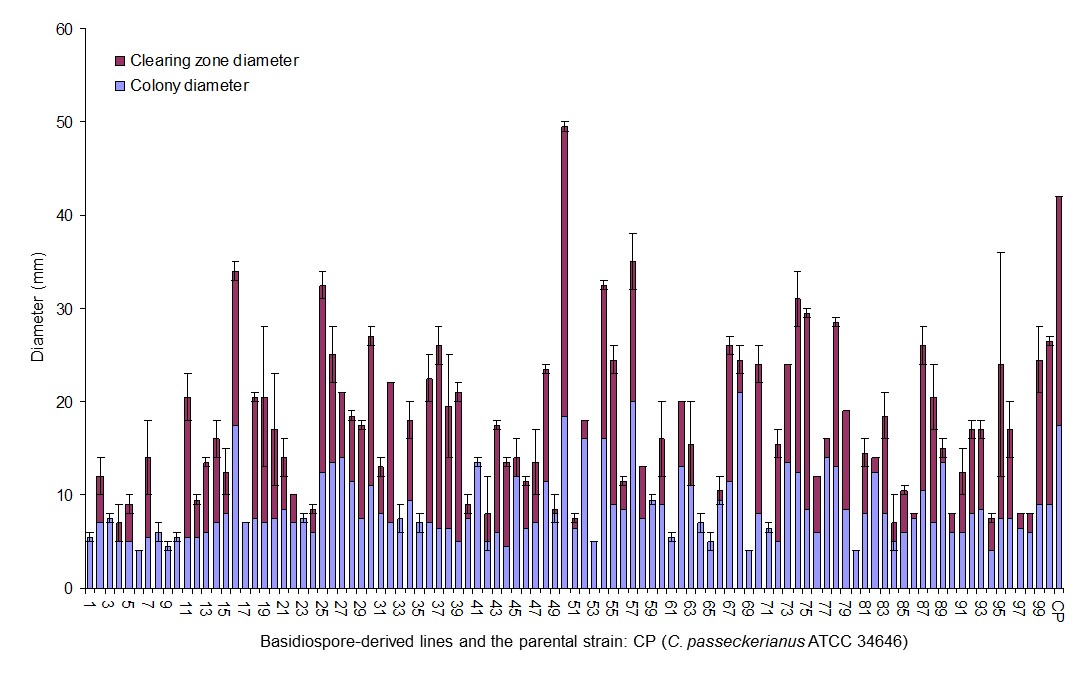


**Supplementary Figure 3**: The clearing zone areas observed for bioassay plates of 100 basidiospore lines and CP1, a dikaryotic strain of *Clitopilus passeckerianus*. Standard error bars are shown. Numbers 22, 27 and 76 have no standard error bars because only one result was obtained (due to contamination). All others with no standard error bars are due to the replicates giving identical results.


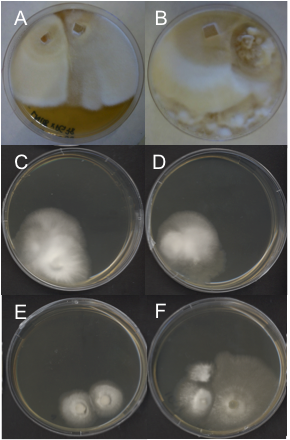


**Supplementary Figure 4**: A and B: *Coprinopsis cinerea* controls for mating crosses. An incompatible pairing of strains LN118 and PG78 (B) and a compatible mating between LN118 and AT8, where the resulting dikaryon demonstrates an increased growth rate and abundant aerial hyphae. C-F: Examples of mating crosses for *C. passeckerianus*. C and D show a clear change of phenotype including increased aerial hyphae, originating from the point of contact for the two colonies. E is a negative control where the plate has been inoculated twice with the same strain. F shows an apparent phenotypic change but is lacking the characteristic increased aerial mycelia and growth rate.

##
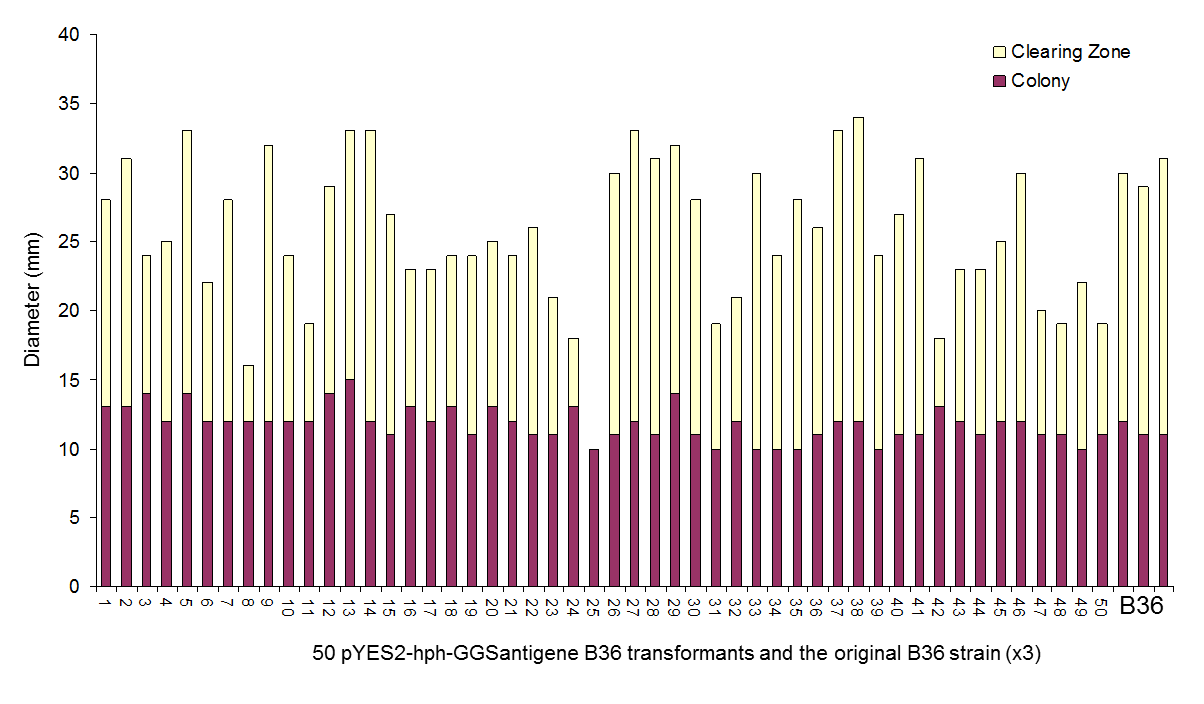


**Supplementary Figure 5**: Bioassays to assess pleuromutilin production in B36 transformants containing pYES-hph-GGSantigene, a plasmid designed to silence the pleuromutilin GGS.

## Supplementary Tables

**Supplementary Table 1**: Percentage sequence identity, at the protein level, for the predicted homeodomain transcription factors of *Clitopilus passeckerianus* ATCC 34646. Proteins beginning HDI are located in allele 1 of the HD locus, HD2 proteins are located in allele 2. ‘hdt1’ refers to the identity of the protein as a type 1 homeodomain transcription factors and ‘hdt2’ are type two homeodomain transcription factors.

|  | **HD1-hdt2a** | **HD1-hdt1b** | **HD1-hdt2b** | **HD1-hdt1c** | **HD1-hdt2c** | **HD2-hdt1a** | **HD2 hdt2a** | **HD2-hdt1b** | **HD2-hdt2b** | **HD2-hdt1c** | **HD2-hdt2c** |
| --- | --- | --- | --- | --- | --- | --- | --- | --- | --- | --- | --- |
| **HD1-hdt2a** | - |  |  |  |  |  |  |  |  |  |  |
| **HD1-hdt1b** | 10 | - |  |  |  |  |  |  |  |  |  |
| **HD1-hdt2b** | 17 | 11 | - |  |  |  |  |  |  |  |  |
| **HD1-hdt1c** | 12 | 14 | 9 | - |  |  |  |  |  |  |  |
| **HD1-hdt2c** | 14 | 7 | 9 | 10 | - |  |  |  |  |  |  |
| **HD2-hdt1a** | 9 | 9 | 11 | 10 | 9 | - |  |  |  |  |  |
| **HD2 hdt2a** | 16 | 10 | 11 | 11 | 11 | 8 | - |  |  |  |  |
| **HD2-hdt1b** | 8 | 8 | 8 | 13 | 6 | 7 | 6 | - |  |  |  |
| **HD2-hdt2b** | 10 | 8 | 9 | 9 | 10 | 8 | 9 | 7 | - |  |  |
| **HD2-hdt1c** | 7 | 6 | 7 | 5 | 5 | 7 | 8 | 7 | 11 | - |  |
| **HD2-hdt2c** | 11 | 8 | 10 | 9 | 55 | 8 | 11 | 3 | 9 | 5 | - |
